# Quantification of Etoricoxib in Low Plasma Volume by UPLC-PDA and Application to Preclinical Pharmacokinetic Study

**DOI:** 10.3390/ph17040507

**Published:** 2024-04-16

**Authors:** Sapir Ifrah, Daniel Porat, Mordechai Deutsch, Arik Dahan

**Affiliations:** 1 Department of Clinical Pharmacology, School of Pharmacy, Faculty of Health Sciences, Ben-Gurion University of the Negev, Beer-Sheva 8410501, Israel; sapirgar@post.bgu.ac.il (S.I.); poratdan@post.bgu.ac.il (D.P.); 2 Department of Nephrology and Hypertension, Meir Medical Center, Kfar Saba 4428164, Israel; motti.jsc@gmail.com

**Keywords:** liquid–liquid extraction, COX-2 inhibitor, etoricoxib, trazodone, analytical method, drug determination, chromatography, mini-capsule

## Abstract

An ultra-performance liquid chromatography with photodiode array (UPLC-PDA) UV detection method was developed here for the first time for simple, rapid, selective and sensitive quantification of the commonly prescribed selective cyclooxygenase-2 (COX-2) inhibitor etoricoxib in low plasma volumes (50 μL). The method includes protein precipitation followed by liquid–liquid extraction, evaporation and reconstitution. A gradient mobile phase of 75:25 going to 55:45 (*v*/*v*) water:acetonitrile (1 mL/min flow rate) was applied. Total run time was 8 min, representing a significant improvement relative to previous reports. Excellent linearity (r^2^ = 1) was obtained over a wide (0.1–12 µg/mL) etoricoxib concentration range. Short retention times for etoricoxib (4.9 min) and the internal standard trazodone (6.4 min), as well as high stability, recovery, accuracy, precision and reproducibility, and low etoricoxib LOD (20 ng/mL) and LOQ (100 ng/mL), were achieved. Finally, the method was successfully applied to a pharmacokinetic study (single 20 mg/kg orally administered etoricoxib mini-capsule) in rats. In conclusion, the advantages demonstrated in this work make this analytical method both time- and cost-efficient for drug monitoring in pre-clinical/clinical settings.

## 1. Introduction

Etoricoxib is a selective cyclooxygenase-2 (COX-2) inhibitor, approved in more than 80 countries worldwide and indicated for the treatment of rheumatoid arthritis, psoriatic arthritis, osteoarthritis, ankylosing spondylitis, chronic low back pain, acute pain and gout. At its maximal single unit dose, 120 mg, it was shown to be at least as effective as other analgesics commonly used [1]. As a highly selective COX-2 inhibitor, its use is involved with significantly fewer gastrointestinal adverse effects compared to non-selective NSAIDs [2]. The drug is orally administered, exhibiting complete absorption with 100% bioavailability. It is extensively metabolized by, mainly, cytochrome P450 3A4 (CYP3A4) to inactive metabolites [3]. Etoricoxib is a lipophilic (logP = 2.8), and weakly basic drug (pKa = 5.0), showing low and pH-dependent aqueous solubility. It is classified as a BCS (biopharmaceutics classification system) class II compound [4].

Etoricoxib was previously quantified in human plasma by several different methods, including high-performance liquid chromatography (HPLC) with post-column photochemical derivatization-fluorescence detection [5], HPLC with ultraviolet (UV) detection [6,7], HPLC-tandem mass spectrometry (MS/MS) [8], liquid chromatography (LC)-MS/MS with electrospray ionization [3,9] and ultra-performance liquid chromatography (UPLC) with MS/MS [10]. Etoricoxib was also quantified in rat plasma by means of LC-MS [11,12] and HPLC-UV [13]. The LC-MS/MS tool is both fast and sensitive [10,14]; however, this advanced equipment is only affordable for some laboratories. Meanwhile, due to relatively low costs, UPLC with UV (UPLC-UV) or, preferably, photodiode array (UPLC-PDA) detection, is affordable, and can allow rapid analysis with good sensitivity [15,16]. Yet, the use of UPLC-PDA to determine etoricoxib in either human or rat plasma has not been previously investigated. As for etoricoxib, it is available in fixed-dose combinations with other drugs, including paracetamol, thiocolchicoside, toperisone and pregabalin, emphasizing the need for a simple and reliable method for the separation and quantification of this clinically important anti-inflammatory agent.

Although etoricoxib levels are not regularly monitored in patients, quantifying this drug in plasma may be important for various applications, including bioequivalence tests for developing generic products, developing new formulations, and in drug repurposing research. In bioequivalence studies, the active ingredients’ absorption rate and extent are investigated from generic preparations relative to the innovative product; such a study by Meulman et al. tested the performance of etoricoxib-coated tablets in healthy volunteers [17]. In the development of new formulations of etoricoxib, many factors must be considered to ensure the safety, efficacy, and stability of the new product, and blood levels must be determined through clinical trials. For instance, Sapkal et al. developed a solid dispersion of etoricoxib using natural polymers for solubility/dissolution enhancement [18]. These new formulations are not limited to the oral route, e.g., injectable formulations for the treatment of osteoarthritis, an existing indication of etoricoxib [19,20]. Drug repurposing is one of the most promising strategies to broaden our treatment options in various medical conditions, with many examples of the successful therapeutic switching of old molecules to new pathways [21]. Specifically, for the coxibs family, clinical potential in metronomic chemotherapy, the treatment of mental disorders, or infectious diseases have been discussed [22]. Etoricoxib was recently studied for its clinical efficacy in lung cancer [23] and glioblastoma cells [24]. Etoricoxib was also investigated in patients for the treatment of incident hypoxia [25] and was found to prevent weight gain in rodents [26]. Throughout these repurposing studies, and certainly in any future development of new drug products, the quantification of etoricoxib in plasma is an essential step. 

In this study, we developed and validated a simple, rapid, and accurate UPLC-PDA method for the determination of etoricoxib in low plasma volumes (50 μL). Then, we successfully applied this analytical method to a pharmacokinetic study of etoricoxib (single 20 mg/kg dose) after the oral administration of a mini-capsule to Sprague Dawley rats. 

## 2. Results

### 2.1. Chromatography

**Figure 1:**
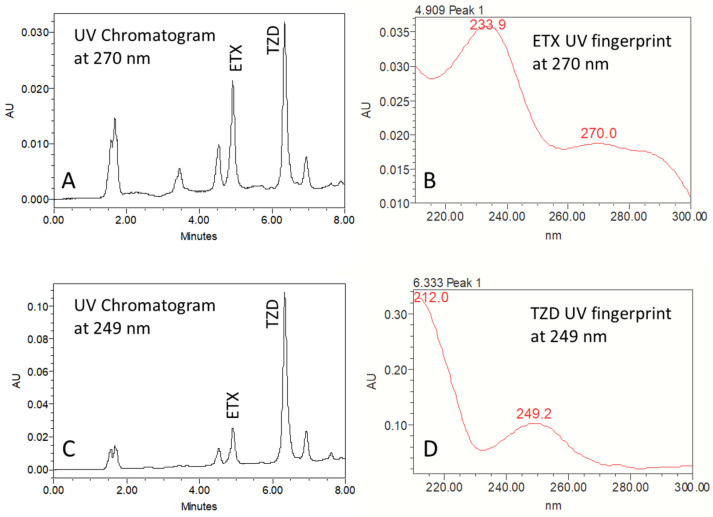
Ultraviolet chromatograms at 270 nm (**A**) and 249 nm (the wavelength used for the internal standard trazodone HCl quantification) (**B**), showing the separation of the eluted etoricoxib (ETX) and trazodone HCl (TZD) in plasma, reproduced from one of the pharmacokinetic study samples; ultraviolet fingerprints of ETX at 270 nm (**C**) and of TZD at 249 nm (**D**).

The final method was obtained following several trials with different solvents, internal standards and mobile phase gradients. The finally adopted gradient mobile phase consisted of 75:25 up to 55:45 (*v*/*v*) water:ACN (0.1% TFA in both). The detected UV wavelength was 270 nm for etoricoxib and 249 nm for the IS. A chromatogram for etoricoxib and IS in rat plasma is presented in Figure 1 and characterized by good separation. The LOD and LOQ for etoricoxib were determined as 20 and 100 ng/mL, respectively. A short 4.9 min elution time for etoricoxib, and 6.4 min for the IS, were obtained using this method (Figure 1). This is a significant improvement over previous HPLC-UV assays of etoricoxib in rat plasma, eluting the drug at 10 [13] or 15.6 min [7].

### 2.2. Method Validation

**Figure 2:**
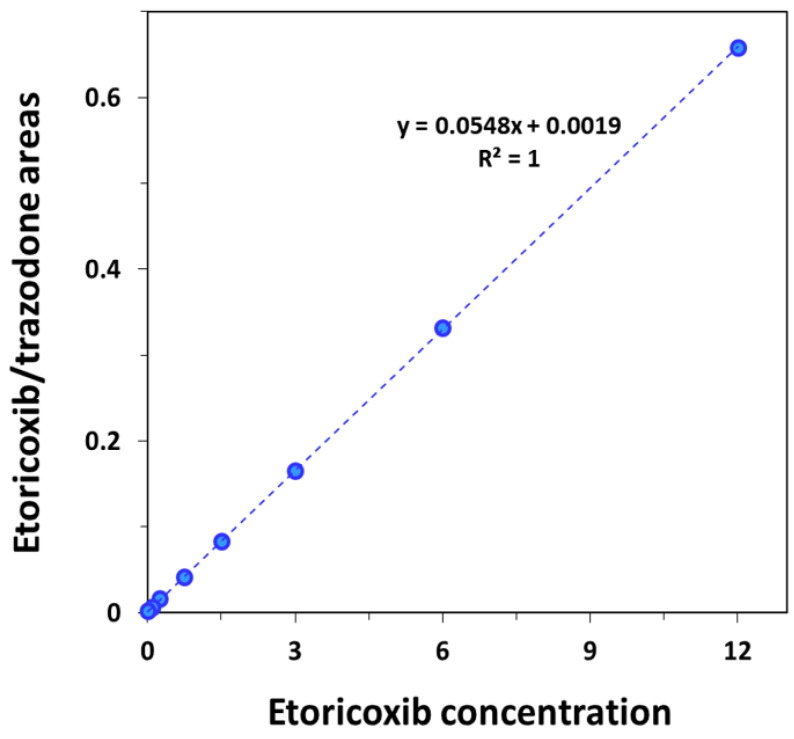
Calibration curves of blank rat plasma spiked with eight different etoricoxib plasma concentrations. The curve was constructed by plotting the area ratio of etoricoxib to internal standard vs. etoricoxib concentration.

Selectivity was determined by analysis of spiked plasma samples from six different rats, in which no interference was seen in the retention times of the analyte and IS. The matrix effect showed the low interference of 11% from the sample matrix. That is, there is no measurable analyte in the matrix and the matrix effect is low; therefore, there is no effect on the results of the bioanalysis method validation. Stability was confirmed for all studied samples (*n* = 3) and concentrations (12 and 0.1 µg/mL) after 4 h at room temperature (95.2% and 110%, respectively) and after 24 h at 4 °C (91.1% and 99.2%, respectively). This is in line with the available literature on etoricoxib being stable in plasma under different conditions [10]. Linearity was determined by analyzing a series of calibration standards of blank plasma spiked with eight different concentrations (including 0 µg/mL) of etoricoxib. The curve was constructed with the *x*-axis as the concentration of etoricoxib and the *y*-axis as the ratio between the areas of etoricoxib and the IS. The curve at concentrations 0–12 µg/mL was linear (R^2^ = 1) (Figure 2). Accuracy and precision were studied via the injection of six replicates of three different concentrations of 0.1, 3 and 12 µg/mL. The means (*n* = 6) of intra- and inter-day accuracy values of three different concentrations were <5% and <1%, respectively, while intra- and inter-day precision were <3% and <5%, respectively (Table 1). The efficiency of extraction recovery from rat plasma-spiked etoricoxib concentrations 3 and 12 µg/mL were 90.0 ± 0.55 and 102.1 ± 0.12%, respectively.

### 2.3. Pharmacokinetic Study

**Figure 3:**
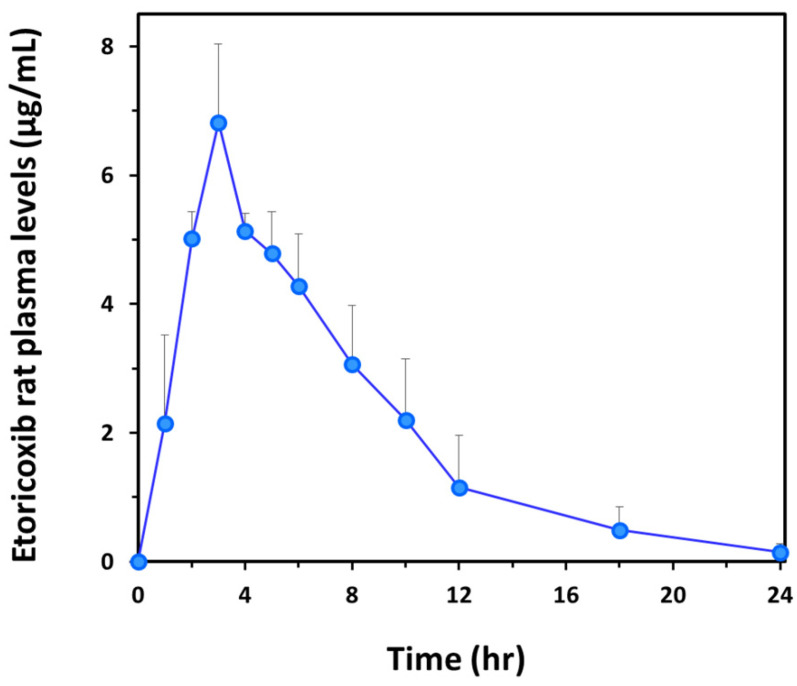
The mean plasma concentration–time curve of etoricoxib in rats (*n* = 3) after a single intragastric administration of 20 mg/kg of the drug inside a mini-capsule. Bars represent standard deviation.

The analytical method was applied to a pharmacokinetic study in rats (*n* = 3) following a single intragastric administration of a 20 mg/kg etoricoxib mini-capsule. The mean plasma concentration–time curve is presented in Figure 3. The main pharmacokinetic parameters were obtained using the PKSolver 2.0 software. Mean (SD) *C*_max_ and *AUC*_0–*t*_ were 6.8 (1.2) µg/mL and 48.9 (13.0) μg × h/mL, respectively. The *t*_max_ of all three rats was 3 h, and the mean *t*_1/2_ was 3.7 (1.0) (Table 2). 3 h after administration, etoricoxib was steadily removed from plasma, showing significant plasma concentrations after 12 h (~1 µg/mL) and detectable levels after 24 h (~0.2 µg/mL).

## 3. Discussion

In this study, we were able to develop a simple and economic analytic method for the rapid, accurate and reliable plasma quantification of the COX-2 inhibitor etoricoxib, with successful application to an in vivo pharmacokinetic study. This analytic method was validated successfully, with short etoricoxib retention time, low limit of quantification (LOQ) and high recovery. The LOQ was lower than the plasma etoricoxib levels at all time points (for all three rats), including 24 h, despite the fact that etoricoxib was administered in a solid dosage form, rather than the more common form in rats—liquid. The above indicates the applicability of this analytic method to the pharmacokinetic study.

The pharmacokinetic profile of each rat was fairly similar, especially in the absorption phase, with the same *t*_max_ of 3 h and comparable maximal concentrations (*C*_max_). Steady elimination was observed in all three rats, with slightly different rates (Table 2). No single outlier concentration was obtained during the pharmacokinetic analysis of each rat, and the same was true for the mean values (Figure 3), despite the challenging, variability-prone mini-capsule oral dosing, suggesting that the bioanalysis is reliable and can be successfully employed in a pharmacokinetic study. In addition, the dose administered (20 mg/kg) translated into an overall plasma exposure that was comparable to that in humans after the oral administration of a single 120 mg drug tablet (*AUC*_0–*t*_ ~ 50 μg∙h/mL) [27]. In addition, drug levels in patients after 120 mg etoricoxib were around 1 µg/mL, well above the LOQ for this method to be sufficiently sensitive to capture that information [10,28]. In previously established methods for quantifying etoricoxib in plasma, the reported LOQs were lower than here, including 50 ng/mL (HPLC-UV) [29] and 0.004 µM (~1.4 ng/mL; LC-MS/MS) [11] using rat plasma. To note, in the reported HPLC-UV method, the plasma samples’ volumes were 0.5 mL, which is 10-fold higher than here (50 µL).

The oral drug administration of a solid dosage form to rats is both uncommon and challenging in terms of the handling technique and the special equipment required, which demands proficiency and experience. Such dosage forms may also result in lower, or potentially suboptimal, plasma drug levels (in terms of bioanalysis) due to incomplete disintegration/dissolution, lowering the rate and extent of oral drug absorption. Despite all of the above, we chose to administer the drug as a capsule, because solid oral drug administration is clinically far more common, making the procedure closer to treatment in humans, and more applicable for human pharmacokinetics. Indeed, etoricoxib is only commercially available in the form of solid tablets. Especially in this case, since this drug has low and pH-dependent solubility, choosing a liquid dosage form for a pharmacokinetic study of etoricoxib may mask the solubility/dissolution factor, leading to higher drug levels; indeed, compared to our results, Balap et al. administered the drug in a liquid and reported much higher etoricoxib plasma levels, with almost 5-fold higher *AUC*_0–24_ and 2.7 times higher *C*_max_, and this is despite a lower single dose of 10 mg/kg (compared to the 20 mg/kg dose in our study). These results can be attributable to the differences in dosage form. Following capsule administration, disintegration and de-aggregation must first occur before drug dissolution may take place. This can both prolong and even hinder the absorption and overall exposure of low-solubility drugs such as etoricoxib [30]. Still, the elimination phase of etoricoxib, which is not related to the dosage form but to the drug entity and the rat physiology, was very similar in both our study with the mini-capsule and the study of Balap et al. using the liquid dosage form, affirming the attribution of the absorption differences to the dosage form.

The Thermo Hypersil BDS column was chosen as it is suitable for the quantification of basic lipophilic compounds, such as etoricoxib. Plasma alkalization with 0.1 M NaOH was performed to decrease the solubility of the studied drug and IS (both weakly basic) in the aqueous phase, improving the extraction and selectivity towards the organic solvent. Non-maximal detection wavelengths of 270 and 249 nm were chosen as they involve stable, low-variance absorption and are unique for the drug and IS, allowing high assay sensitivity. Trazodone HCl (pKa ~ 7, logP ~ 3) was chosen as the internal standard because of its roughly similar physicochemical properties to etoricoxib, both being weakly basic and lipophilic, allowing effective simultaneous extraction, and importantly, the two compounds were found to comfortably elute 1 min apart from each other (Figure 1). This compound is also highly available and affordable. An initial attempt was made with lamotrigine as an internal standard. However, lamotrigine did not separate well from etoricoxib. In fact, with the developed method, an internal standard characterized by earlier elution from a C18 reversed-phase column can be used to achieve a run-time as short as 6 or even 5 min. This can be useful for the analysis of a large number of samples when performing serial dosing in larger-scale studies or in cases of multiple/chronic dosing.

Despite the small number of animals in the pharmacokinetic study, the similar, low variance and logical pharmacokinetic profiles (not shown) and parameters (Table 2) among the three rats indicate that the developed method can be applied with high probability in a pharmacokinetic experiment.

In this study, we show a reliable and accurate method for the quantification of etoricoxib in particularly small plasma volumes. For example, in a previous study quantifying etoricoxib in rat plasma, larger plasma volumes (150 μL) were used for the LC/MS analysis [11]. This can allow more frequent blood sampling, as well as the extension of the pharmacokinetic experiment from a single-dose to a multiple-dosing regimen. Another advantage of this method is the use of low-volume organic solvents, making the technique both affordable and easily applicable, with most steps of the sample preparation occurring within a single 1.5 mL Eppendorf vial. Indeed, previous studies used larger volumes of organic solvents to extract the drug [7,11,13]. 

Overall, this simple and selective UPLC-PDA analytical method is suitable for the accurate quantification of etoricoxib using small volumes (50 µL) of plasma obtained during a pharmacokinetic study. The advantages demonstrated in this work make this analytical technique both a time- and cost-efficient approach for etoricoxib monitoring in the pre-clinical/clinical laboratory.

## 4. Materials and Methods

### 4.1. Chemicals and Reagents

Etoricoxib and trazodone HCl were purchased from Sigma-Aldrich. Ethyl acetate and trifluoroacetic acid (TFA) were purchased from Sigma Chemical Co. (St. Louis, MO, USA). Acetonitrile (ACN) and double-distilled water (Merck KGaA, Darmstadt, Germany) were of UPLC grade. All other chemicals were of analytical reagent grade.

### 4.2. Stocks and Working Solutions

Etoricoxib and trazodone HCl (internal standard) were each dissolved in ACN to prepare 600 µg/mL stock solutions. A working solution for the internal standard (IS) was prepared from the stock solution by dilution with ACN to 12 μg/mL. To construct the calibration curve and validation tests, further dilutions of the etoricoxib stock solution with ACN were made.

### 4.3. Equipment

Etoricoxib analysis was performed by UPLC on a Waters (Milford, MA, USA) Acquity UPLC H-Class system equipped with Photodiode-Array Detection (PDA) and Empower 2 software (Milford, MA, USA). Etoricoxib and trazodone HCl (IS) were analyzed using a Thermo Hypersil^®^ BDS C18 5 μm, 150 × 4.6 mm column (Thermo Fisher Scientific, Passau, Germany). The mobile phase contained 0.1% TFA in water and 0.1% TFA in ACN; a gradient flow was used, going from a 75:25 to a 55:45 ratio over 6 min. The flow rate was 1 mL/min, and the injection volume was 70 μL. The total run time was 8 min. The peak areas of etoricoxib and trazodone HCl were determined at wavelengths of 270 and 249 nm, respectively. Ambient temperature was used for both the column and the samples.

### 4.4. Calibration Curve

Etoricoxib stock solution was diluted to obtain the following concentrations: 60, 30, 15, 7.5, 3.75, 1.25 and 0.5 μg/mL. Then, inside 1.5 mL Eppendorf tubes, blank rat plasma samples of 40 μL were spiked with 10 μL of the different concentrations of etoricoxib in ACN to obtain 12, 6, 3, 1.5, 0.75, 0.25 and 0.1 μg/mL of etoricoxib in plasma medium, respectively; 10 μL of 0.1 M NaOH was then added for plasma alkalization. Next, plasma protein was precipitated by the addition of 100 μL IS working solution (12 μg/mL trazodone HCl in ACN), followed by a 1 min vortex. Then, liquid–liquid extraction was performed using 1 mL ethyl acetate, followed by a 1 min vortex and a maximum-speed centrifuge 14,000 rpm (20,817 rcf) at 5 °C for 10 min; 850 μL of the organic phase supernatant was moved to glass tubes for evaporation using a multiple-sample evaporator (Labconco RapidVap^®^, Kansas City, MO, USA) at 37 °C with 30% vortex motion and a vacuum level of 100 mbar for 15 min. Following evaporation, the dried sample was reconstituted with 150 μL of 1:1 water:ACN and filtered through a 0.22 µm PTFE hydrophilic filter (Membrane Solution, LLC, Auburn, WA, USA). Finally, 70 μL was injected into the UPLC system.

### 4.5. Method Validation

The analytical method was validated for selectivity, linearity, precision, accuracy, matrix effects, recovery, and stability. Briefly, selectively was validated by examination of blank plasma samples from six individual rats, spiked with analyte. Linearity was determined by the concentration of analyte vs. the ratio of the peak area of the analyte to the internal standard when the calibration curve presented a linear model 1/x^2^ weighted linear least-squares regression model. The determined limit of quantification (LOQ) is the lowest drug concentration validated with an accuracy and precision lower than 20%. The determined limit of detection (LOD) is the lowest drug concentration that can be detected by UPLC with an accuracy and precision lower than 20%. Accuracy is defined by the percentage relative error (%RE):RE (%) = [(mean measured − spiked)/spiked] × 100 and the precision is determined by intra- and inter-day relative standard deviation. The quality control (QC) solution and etoricoxib standard solution were prepared by the dilution of stock solution with ACN. Intra-day accuracy and precision were determined by analyzing six QC replicates of three concentrations (0.1, 3 and 12 µg/mL) on the same day, whereas inter-day accuracy and precision were determined by analyzing six QC replicates of the same concentrations on three different days. Recovery was evaluated by the ratio of the peak areas from QC samples (0.1, 3 and 12 µg/mL) to those obtained from the analyte spiked into an equivalent volume of post-extraction supernatant. Matrix effect was determined by the ratio of peak areas from post-extraction samples to that from an equivalent concentration of the pure standard solution. Stability was evaluated under different conditions applied to rat plasma samples containing low (0.1 µg/mL) and high (12 µg/mL) etoricoxib concentrations, including 4 h at room temperature and 24 h at 4 °C.

### 4.6. Pharmacokinetic Study

Our developed method was applied to a rat pharmacokinetic study (*n* = 3), with single-dose etoricoxib administered orally at a dose of 20 mg/kg. Sprague-Dawley male rats were supplied by Harlan Laboratories Ltd., Jerusalem, Israel, and housed and handled according to the Ben-Gurion University of the Negev Unit for Laboratory Animal Medicine Guidelines. All animal protocols were approved by the Animal Use and Care Committee of the Ben-Gurion University of the Negev, Beer-Sheva, Israel (Approval number IL-31-05-2021D). 

Two days before the pharmacokinetic study, rats underwent jugular vein cannulation under ketamine–xylazine anesthesia. A 1 cm longitudinal incision was made on the skin over the jugular vein, and the vein was exposed by clearing the surrounding soft tissues. At this point, while the shorter end of the cannula was out of the jugular vein, a sterile disposable syringe containing heparinized (20 U/mL) normal saline for injection was connected to the longer end of the silicone cannula by a blunt-ended 23-gauge sterile needle. The implementation needle was disconnected, and the cannula was flushed again with normal saline. Then, the short end of the cannula was slowly passed into the jugular vein. The cannula was then fixed in place by suturing the silk thread to the muscle. The muscle at the incision was closed, followed by the open skin.

Rats were fasted for 12 h before the pharmacokinetic study, with free access to water. On the day of the study, size 9 mini-capsules (Torpac^®^ Inc., Fairland, NJ, USA) were filled with 20 mg/kg etoricoxib and administered to the stomach using a special capsule-holder and dosing syringe (Torpac^®^ Inc., Fairland, NJ, USA); 150 µL of the venous blood was collected into 1 µL heparin-containing Eppendorf tubes at fixed times: 1, 2, 3, 4, 5, 6, 8, 10, 12, 18 and 24 h post-dosing. Blood was centrifuged at 4500 rpm (2151 rcf) for 7 min, and plasma was collected and stored at −20 °C until analysis. For analysis, 50 µL of plasma was used, undergoing, simultaneously, the same aforementioned procedure of NaOH plasma alkalization, protein precipitation with ACN containing the internal standard, and ethyl acetate-based liquid–liquid extraction as was used for the calibration curve.

## Figures and Tables

**Table 1:** Intra-day and inter-day precision and accuracy for etoricoxib in rat plasma (*n* = 6).

Spiked Concentration (µg/mL)	Measured Area Overall Mean ± SD (μg∙h/mL)	Intra-Day Precision (%)	Inter-Day Precision (%)	Accuracy (%)
0.1	18,188 ± 859	2.65	4.72	3.97
3	536,753 ± 12,531	1.69	2.33	1.26
12	2,152,069 ± 35,528	0.39	1.57	1.54

**Table 2:** The main pharmacokinetic parameters of etoricoxib following intragastric administration of a single 20 mg/kg etoricoxib mini-capsule to rats (*n* = 3).

Parameter	Description	Mean (SD)	Rat 1	Rat 2	Rat 3
*t*_1/2_	Half-life, h	**3.7 (1.0)**	4.5	2.5	3.6
*t*_max_	Time to reach *C*_max_, h	**3**	3	3	3
*C*_max_	Maximum plasma concentration, µg/mL	**6.8 (1.2)**	5.5	7.9	7.0
*AUC*_0–*t*_	Area under the curve (0–t), μg∙h/mL	**48.9 (13.0)**	51.8	37.7	63.7

## Data Availability

The data presented in this study are available on request from the corresponding author.
